# Real world outcomes with Tepotinib in a series of Indian patients with MET exon 14 skipping positive non-small cell lung cancer

**DOI:** 10.3332/ecancer.2025.1966

**Published:** 2025-08-15

**Authors:** Rohini Sebastian, Josh Thomas Georgy, Arun George, Prisca Santhanam, Raiza Philip, Anjana Joel, Ajoy Oommen John, Deepa Susan Joy Philip, Divya Bala Thumaty, Thomas Alex Kodiatte, Ashish Singh, Rekha Pai

**Affiliations:** 1 Department of Pathology, Christian Medical College, Vellore, TN 632004, India; 2 Department of Medical Oncology, Christian Medical College, Vellore, TN 632004, 632004; * Contributed equally

**Keywords:** METex14, NGS, NSCLC, Tepotinib, targeted therapy

## Abstract

The plethora of targetable variants among non-small cell lung cancers is on the rise, making it one of the most important cancer types in the context of precision oncology. Recently, the MET exon14 skipping mutation has emerged as a novel therapeutic target. This mutation results from somatic alterations at the splice junction of exon 14 of the MET gene, leading to constitutive activation of downstream signaling pathways. The approval of targeted therapy for this variation makes it a compelling need to use appropriate testing systems for detection. Utilising a robust next-generation sequencing platform, we have identified this mutation in 5.3% of cases in our cohort. In the absence of information on MET exon 14 skipping from India, this case series will throw some light on this variation in our subcontinent and highlights the fact that the real-world effectiveness of MET inhibitors like Tepotinib and Capmantinib might be lower than expected.

## Background

Lung cancer epitomises precision oncology, which centers on targeting molecular alterations such as Epidermal Growth Factor Receptor (EGFR), Kirsten Rat Sarcoma Viral oncogene homolog (KRAS) G12C and Anaplastic Lymphoma Kinase (ALK), found in 40%–50% of lung adenocarcinomas. The spectrum also includes less frequent alterations, including RET, ROS1, BRAF and NTRK [1]. The MET exon14 skipping mutation has recently emerged as a novel therapeutic target.

**Figure 1.**
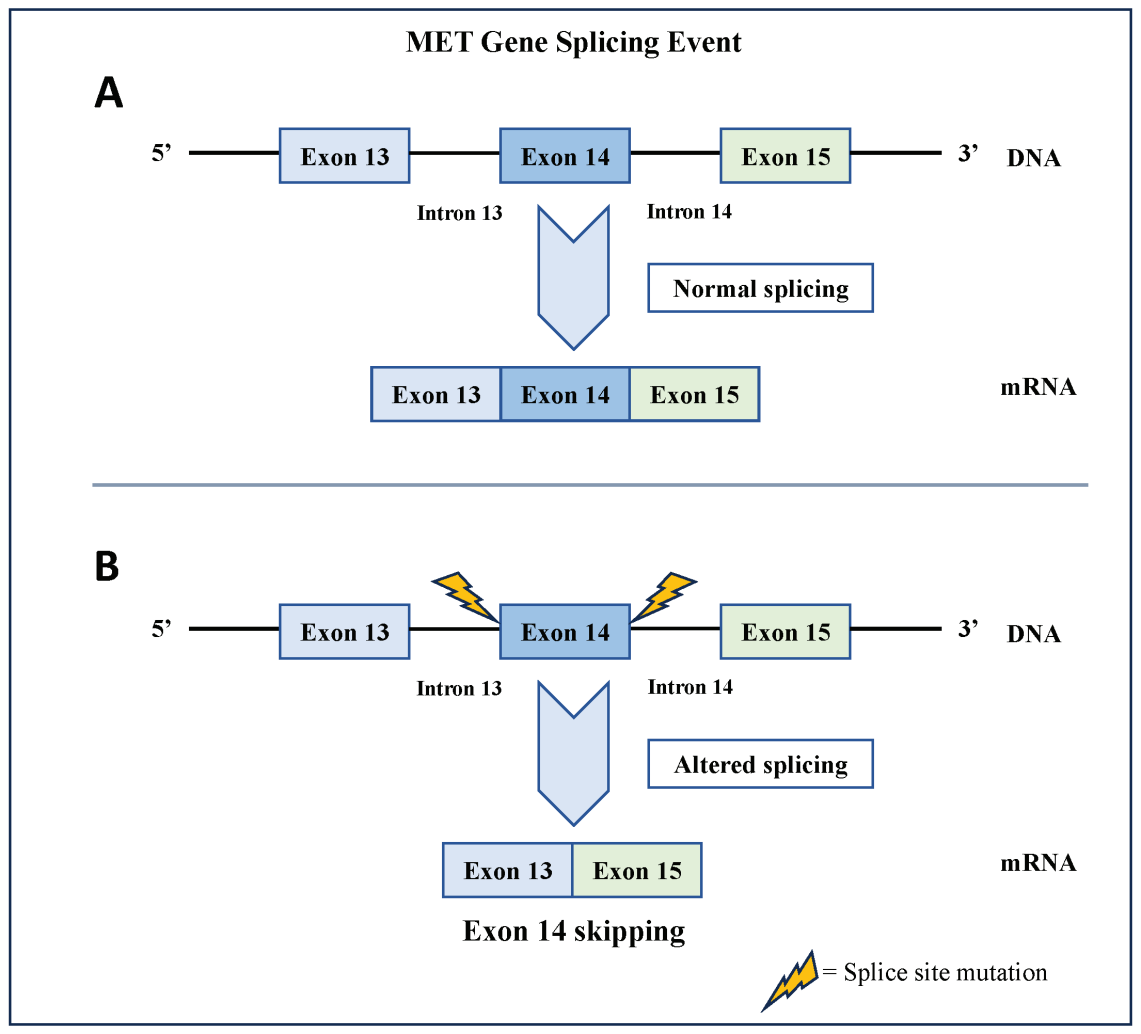
Illustration of the MET gene splicing event. (a): Normal splicing results in an mRNA sequence containing exon 13, exon 14 and exon 15. (b): Splice-site mutations lead to altered splicing, resulting in exon 14 skipping.

The MET gene encodes the MET receptor tyrosine kinase, a crucial regulator of cellular processes like proliferation, survival and motility. Somatic alterations near the splice junction of exon 14 cause its skipping during transcription, resulting in a mature mRNA with fused exons 13 and 15 [2] (Figure 1). T﻿he discovery of MET exon 14 skipping mutations and the development of small-molecule inhibitors emphasize the importance of molecular profiling in non-small cell lung cancer (NSCLC). The Food and Drug Administration's (FDA's) approval of oral tyrosine kinase inhibitors, Capmatinib in 2020 and Tepotinib in 2021, for metastatic NSCLC with MET exon 14 skipping mutations, highlights the need to detect this variation among NSCLC [3]. This study presents the first case series of MET exon 14 skipping mutations in NSCLC patients from the Indian subcontinent, highlighting the treatment course and outcomes from a resource-limited setting.

## Methods

This study was conducted in the molecular pathology laboratory of the Department of Pathology, Christian Medical College, Vellore, Tamil Nadu, India. One hundred and thirty cases diagnosed as NSCLC on histopathology from June 2023 to January 2024, with sufficient tissue for extraction of DNA and RNA in Formalin Fixed Paraffin Embedded (FFPE) blocks, were included for testing by next generation sequencing (NGS). The necessary histopathological data were acquired from the pathology database, and clinical details from the clinical workstation. Four-micron-thick sections were cut from FFPE blocks. DNA and RNA were extracted using MagMAX™ FFPE DNA/RNA Ultra Kit (Thermo Fisher, USA) following the kit instructions. The final elute is quantified using Qubit™ 3 Fluorometer (Invitrogen™, USA). Complementary DNA (cDNA) conversion was done using the NGS Reverse Transcription Kit (Ion Torrent™, USA). DNA and cDNA were amplified, templated into amplicon-based libraries, prepared for sequencing and templated onto the Ion Chip for sequencing using the Ion Chef System, after proper quality checks. The samples were sequenced using the NGS platform (Ion GeneStudio S5 plus, Thermo Fisher, USA) and the data were analysed using the Ion Reporter™ software 5.20. The Oncomine focus NGS panel (Thermo Fisher, USA), designed to explore somatic alterations in 52 actionable genes (including mutations, copy number variations and fusions), was utilised. 500X depth and 5% variant allele frequency/250 read counts were the minimum requirements to identify significant mutations/fusions. All positive cases were orthogonally confirmed by PCR amplification, followed by Sanger sequencing.

## Results

All clinicopathological and molecular data are summed up in Table 1.

### Patient 1

**Figure 2.**
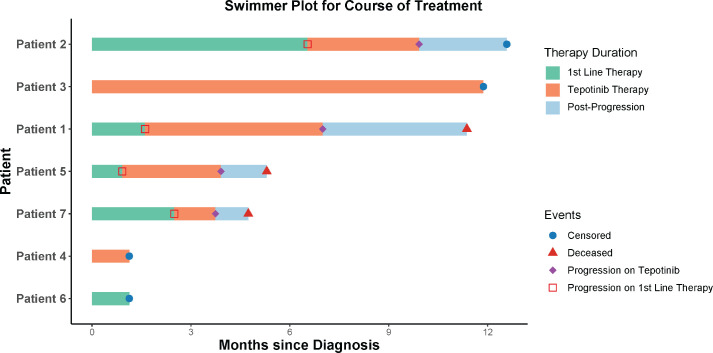
Swimmer plot of treatment course.

**Figure 3.**
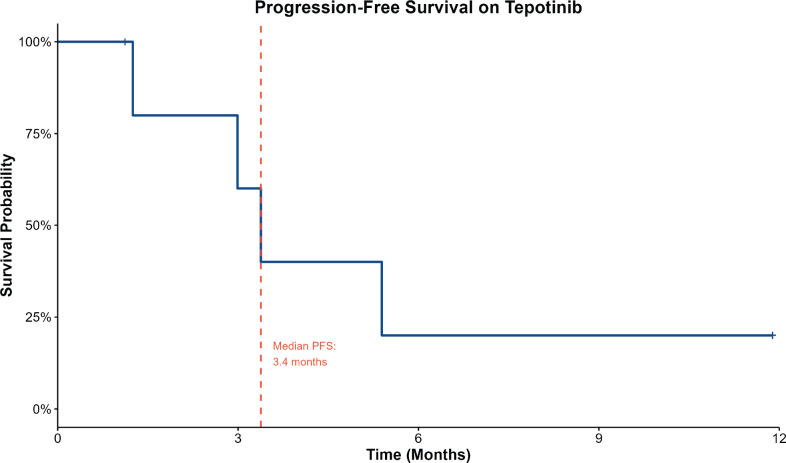
Kaplan–Meier curve of progression-free survival on Tepotinib.

Patient 1, a 61-year-old man with a cavitating lung mass, was diagnosed with keratinising squamous cell carcinoma lung, AJCC TNM stage IIIB. His significant comorbidities included double-vessel coronary artery disease, a history of acute coronary syndrome, diabetes and hypertension, which made him ineligible for concurrent chemoradiotherapy (CRT). He received two cycles of carboplatin, nab-paclitaxel with nivolumab as induction therapy, resulting in stable disease. Treatment was then switched to Tepotinib 450 mg once daily, on which the disease remained stable for 3 months (Figures 2 and 3). On telephonic follow-up, we learnt that the patient discontinued therapy likely due to financial difficulties and/or disease progression and passed away 7 months after stopping Tepotinib.

### Patient 2

Patient 2, a 55-year-old male, presented with bilateral lung nodules, pleural effusion and osseous metastases, diagnosed as stage IVB adenocarcinoma lung with a PD-L1 tumour proportion score of 5%–6% (SP263). He received palliative radiation to the thoracic spine and four cycles of carboplatin, pemetrexed and nivolumab over 3 months, resulting in a partial response. Maintenance therapy with pemetrexed and nivolumab continued for three more months until disease progression. He then began Tepotinib 450 mg once daily, achieving stable disease for 3 months (Figures 2 and 3). During a telephonic follow-up, we learned he was evaluated at another center and, following further progression, is currently receiving third-line therapy with paclitaxel.

### Patient 3

Patient 3, a 70-year-old man with stage IIIB lung adenocarcinoma, was unfit for chemoradiation due to poor ECOG PS and severe COPD. He was initially prescribed 250 mg daily of Gefitinib empirically. After detecting a MET exon 14 skipping mutation, treatment shifted to Tepotinib 450 mg once daily, resulting in a partial response (Figures 2 and 3). During the last follow-up, the disease remained stable and Tepotinib therapy continued. The most significant adverse event was a generalised rash.

### Patient 4

Patient 4, a 59-year-old male with a perihilar mass encasing the aorta and mediastinal lymphadenopathy, was diagnosed with adenosquamous carcinoma of the lung stage IIIC. Due to his comorbidities, and poor ECOG PS, he was unfit for sequential or concurrent chemoradiation and was started on Tepotinib 450 mg once daily (Figures 2 and 3). His last outpatient visit was 1 month post-therapy initiation, after which he was lost to follow-up.

### Patient 5

Patient 5, a 70-year-old woman, presented with an upper lobe lung mass with mediastinal infiltration and lymphadenopathy, diagnosed as squamous cell carcinoma lung, stage IIIC. Due to severe COPD, she received chemoimmunotherapy with two cycles of carboplatin and nivolumab initially. After the NGS report revealed a METex14 mutation, Tepotinib 450 mg once daily was prescribed due to poor tolerance to initial chemoimmunotherapy (Figures 2 and 3). A telephonic follow-up revealed that she had continued Tepotinib for 3 months before stopping due to worsening symptoms and passed away 1 month after discontinuation.

### Patient 6

Patient 6, a 46-year-old male, presented with a lung mass and multiple bone and brain metastases, diagnosed as stage IVB adenocarcinoma lung with focal squamous differentiation. Before arriving at our center, he received whole-brain radiation therapy elsewhere. We began treatment with carboplatin and paclitaxel. After two chemotherapy cycles, reassessment imaging was planned, with the intent to switch to Tepotinib or Capmatinib if progression occurred (Figures 2 and 3). The patient was later lost to follow-up.

### Patient 7

Patient 7, a 61-year-old man, presented with malignant epidural spinal cord compression at the L1 level. Investigations revealed an upper lobe lung mass with hepatic and osseous metastases consistent with stage IVB carcinoma lung. Histopathology showed an adenosquamous carcinoma with 70% PD-L1 expression. We administered palliative spinal radiation followed by 4 cycles carboplatin, paclitaxel and nivolumab. Reassessment imaging showed progressive disease, leading to a switch to Tepotinib 450 mg once daily (Figures 2 and 3). The patient discontinued Tepotinib after 2 weeks due to symptomatic progression and passed away a month later.

### Discussion

The discovery of METex14 mutations has expanded the landscape of targetable mutations in lung cancer. This unique molecular alteration represents a distinct subset of NSCLC, with implications for both prognosis and therapeutic strategies [1, 2]. Our series provides a comprehensive overview of the prevalence, clinical characteristics, treatment course and outcomes in seven Indian patients with locoregionally advanced or metastatic NSCLC harboring METex14, focusing on the unique challenges encountered in a resource-limited setting.

We analysed 130 NSCLC cases diagnosed between June 2023 and January 2024 who underwent NGS-based testing to detect METex14 mutations. The frequency of METex14 mutations in our cohort was 5.4%, which is slightly higher than that reported in previous studies, which generally range from 3% to 4% [2, 4]. This frequency is comparable to and marginally higher than the prevalence of the other non-EGFR actionable oncogenic driver mutations observed in NSCLC namely ROS1 (1%–2%), NTRK1/2/3 (1%), RET (1%–2%), BRAF (1%–5%) and ALK (5%–7%) [5, 6]. The observed prevalence in our cohort raises the possibility that this mutation may be slightly more common in the Indian population compared to global data, possibly due to genetic or environmental factors unique to our demographic.

There was a male preponderance in this study, with 6 of 7 patients being male. This feature has been described by others, including Wang *et al* [9] and Yuan *et al* [8]. Fujino *et al* [7] have also described unique clinical traits in NSCLC patients with METex14 skipping mutations, including older age and smoking history, compared to those with other driver mutations [7]. These characteristics of older age, smoking history and male predominance perhaps reflect trends that can only be verified when larger cohorts are characterised to explore population variability.

We identified the METex14 mutation in various histological subtypes, with adenosquamous carcinoma being the most common in our cohort. Literature shows sarcomatoid carcinoma has the highest prevalence of these mutations, followed by adenosquamous carcinoma, adenocarcinoma and squamous cell carcinoma [2, 10]. The presence of MET exon 14 skipping across histological subtypes underscores the importance of molecular testing among NSCLCs, irrespective of histomorphological features.

METex14 mutations typically occur independently of other driver mutations in NSCLC. Song *et al* [11] identified concurrent EGFR and BRAF mutations in 3 out of 45 NSCLC cases. Schrock *et al* [12] reported concurrent KRAS and EGFR mutations in about 3%–6.4% of cases, respectively. In our series, no concurrent mutations were seen, perhaps explained by the limited numbers included.

Initial treatment regimens in our cohort included combinations of a platinum with either a taxane or pemetrexed, with or without nivolumab, shifting to Tepotinib at progression or upon mutation detection. Most patients had significant comorbidities or poor ECOG PS, precluding aggressive locoregional treatments like surgery or concurrent CRT. In this context, MET inhibitors became a viable option, especially when standard chemotherapy and immunotherapy were contraindicated or poorly tolerated. The efficacy of MET inhibitors varied, reflecting disease heterogeneity and differences in our patients' age, performance status and general health. Patient 3, who was initiated on Tepotinib upfront, had the longest duration of response, which is ongoing at more than 1 year of follow-up (Figure 2). Our real-world data shows lower response rates compared to the 40%–50% ORR reported in the VISION, GEOMETRY and other trials [13, 14]. The median progression-free survival of 3.4 months in our cohort was notably shorter than the PFS reported in those trials. Tepotinib was generally well-tolerated, with one patient reporting a generalised rash and no significant peripheral edema.

The main challenges in our setting were accessibility to Tepotinib/Capmatinib and patient loss to follow-up. In India, patients often travel thousands of kilometres to access tertiary cancer care, making regular follow-up visits difficult. Access to many targeted therapies is limited in LMICs like India due to high costs. Most cancer treatment expenses are out-of-pocket for patients in the Indian healthcare system [15]. With access to essential anti-cancer medicines below 70%, the situation is worse for targeted agents for rare alterations like METex14 [16].

Hence, even at centres where NGS is available to detect the presence of METex14 alteration, treatment is initiated with cytotoxic chemotherapy with or without immunotherapy [17]. Financial constraints and logistical challenges prevent many of our patients from continuing these potentially beneficial treatments and, in some cases, from continuing to seek care altogether. Addressing these barriers is crucial for improving outcomes for patients with METex14-positive NSCLC.

## Conclusion

This study represents the largest series of METex14 NSCLC cases from a single tertiary care center in India, highlighting the unique clinicopathologic and molecular characteristics of this mutation in our population. Although MET inhibitors like Tepotinib and Capmatinib show promise, their real-world effectiveness in our cohort was lower than in global trials. Despite these issues, we advocate for including METex14 in first-line diagnostic panels to guide personalised treatment strategies that could improve patient survival and quality of life.

## Conflicts of interest

There is no conflict of interest to declare.

## Funding

There was no funding received for this study.

## Patient consent statement

The Institutional Review Board provided a waiver of consent as the case series was purely retrospective and fully anonymised.

## Author contributions

RS- generated lab data, collated the lab data with clinical information, prepared manuscript; JTG- helped to recruit cases, contributed to collating lab information with clinical data, prepared manuscript; PS- helped with the lab work; TK, AG, RP- helped with the histopathological workup; AJ, AOJ, DBT, DSJP; AS- helped with the recruitment, treatment, follow up and contributed to the manuscript; RP- Analysed lab data, collated it with clinical information, helped with manuscript writing and editing.

## Figures and Tables

**Table 1.** Clinicopathologic and molecular features.

Patient ID	Age/Sex	Smoking history	Clinical AJCC TNM stage	Histopathology
1	61/M	+	T4N2M0	Squamous cell carcinoma
2	55/M	−	T4N1M1c	Adenocarcinoma
3	70/M	+	T3N2M0	Adenocarcinoma (micropapillary pattern)
4	59/M	+	T4N3M0	Adenosquamous carcinoma
5	70/F	−	T4bN3M0	Squamous cell carcinoma
6	46/M	−	T4N2M1c	Adenosquamous carcinoma
7	61/M	+	T4N3M1c	Adenosquamous carcinoma
Molecular features Variant: MET (13) – MET (15) Locus: chr7:116411708(exon13)-chr7:116414935(exon15) Read count(average): 28225 reads Variant type: Fusion Variant Class [AMP]: IA Variant Class [ACMG]: Pathogenic				
